# Heritability of Malaria in Africa

**DOI:** 10.1371/journal.pmed.0020340

**Published:** 2005-11-08

**Authors:** Margaret J Mackinnon, Tabitha W Mwangi, Robert W Snow, Kevin Marsh, Thomas N Williams

**Affiliations:** **1**School of Biological Sciences, University of Edinburgh, Edinburgh, United Kingdom,; **2**Department of Pathology, University of Cambridge, Cambridge, United Kingdom,; **3**Kenya Medical Research Institute/Wellcome Trust Programme, Centre for Geographic Medicine Research, Coast, Kilifi District Hospital, Kilifi, Kenya,; **4**Nuffield Department of Medicine, John Radcliffe Hospital, Oxford, United Kingdom,; **5**Department of Paediatrics, John Radcliffe Hospital, Oxford, United Kingdom; Royal Melbourne HospitalAustralia

## Abstract

**Background:**

While many individual genes have been identified that confer protection against malaria, the overall impact of host genetics on malarial risk remains unknown.

**Methods and Findings:**

We have used pedigree-based genetic variance component analysis to determine the relative contributions of genetic and other factors to the variability in incidence of malaria and other infectious diseases in two cohorts of children living on the coast of Kenya. In the first, we monitored the incidence of mild clinical malaria and other febrile diseases through active surveillance of 640 children 10 y old or younger, living in 77 different households for an average of 2.7 y. In the second, we recorded hospital admissions with malaria and other infectious diseases in a birth cohort of 2,914 children for an average of 4.1 y. Mean annual incidence rates for mild and hospital-admitted malaria were 1.6 and 0.054 episodes per person per year, respectively. Twenty-four percent and 25% of the total variation in these outcomes was explained by additively acting host genes, and household explained a further 29% and 14%, respectively. The haemoglobin S gene explained only 2% of the total variation. For nonmalarial infections, additive genetics explained 39% and 13% of the variability in fevers and hospital-admitted infections, while household explained a further 9% and 30%, respectively.

**Conclusion:**

Genetic and unidentified household factors each accounted for around one quarter of the total variability in malaria incidence in our study population. The genetic effect was well beyond that explained by the anticipated effects of the haemoglobinopathies alone, suggesting the existence of many protective genes, each individually resulting in small population effects. While studying these genes may well provide insights into pathogenesis and resistance in human malaria, identifying and tackling the household effects must be the more efficient route to reducing the burden of disease in malaria-endemic areas.

## Abstract 2

Genetic factors accounted for one quarter of the total variability in malaria incidence in this study population, slightly less than household-specific environmental factors.

## Introduction

While a growing number of genes have been described that are associated with protection from infection and severe disease due to *Plasmodium falciparum* malaria, the contribution of each gene, or of all the genes combined, relative to the many environmental factors that also influence malarial risk, has rarely been estimated [1,2]. Putting genetic and environmental factors into perspective will inform the design and interpretation of intervention studies aimed at reducing the burden of malarial disease and will help to rationalise research priorities.

It is difficult to estimate the overall contribution of genetic factors (“heritability”) to the between-person variation in the incidence of infectious diseases in the field because it requires both longitudinal data on individual patients sufficient to obtain adequate measures of their risk and also the identification of genetic relatedness between these patients. Furthermore, because related individuals often share a common environment (such as a house), and environmental factors play a major role in the risk of infectious diseases, environmental and genetic effects are inseparable in most field study designs (for example, those that have pairs of full-sibs, each pair living in a different house [1,3–5]). In order to separate these effects, it is necessary to study sets of individuals of varying genetic relatedness who live together in the same house: information on genetically related individuals who live in different households also helps. In this study, we make use of all known genetic relationships within and between houses in order to estimate the heritability of disease risk. This is done, essentially, by regressing the correlation between individuals in their disease incidence on the degree of genetic relationship between them. For example, if the correlation in incidence between full-sibs (who share half their genes) is 0.2, or among half-sibs (who share one quarter of their genes) is 0.1, then the heritability is estimated to be 0.4 [6]. Here we use a generalised version of this principle [7] (the so-called animal model that is widely used in animal breeding) that takes account of all degrees of genetic relatedness to determine the relative contributions of host genetics and other factors to the risk of malaria and other diseases in children living in a malaria-endemic area on the coast of Kenya.

## Methods

### Data Collection

We analysed data from two separate studies, one addressing “mild,” uncomplicated clinical malaria, and the second addressing malaria resulting in admission to hospital, as described in detail previously [8,9]. Briefly, in study 1, the mild disease cohort study, conducted between October 1998 and September 2003, we monitored the incidence of fevers in 640 children 10 y old or younger who were residents of the Ngerenya area of Kilifi District, using active weekly surveillance in the community. The coastal community is made up of a group of nine closely related ethnic groups, broadly known as the Mijikenda, two of which dominated the study populations here. We defined malaria as a measured fever (axillary temperature > 37.5 °C) or a reported history of fever within the preceding 48 h in conjunction with a slide positive for blood-stage asexual *P. falciparum* parasites at any density, and nonmalarial fevers as those in which the blood slide was negative for malarial parasites. We used verbal interview of mothers or key representatives of the household to obtain information on genetic relationships between study children, their parents, and sometimes their grandparents, and to identify genetic links between households (for example, sisters married into different households). From this information, we created a three-column “pedigree” list (one for the individual, two for their parents) of all individuals identified as being related to at least one child in the study. This list was then used to build up a square matrix (“the relationship matrix,” see below) containing coefficients of relationship among all pairs of individuals in the study and among their parents or more distant ancestors where such links were identified [10].

A household typically comprised a group of 3–6 adjacent houses, each occupied by one woman and her children whose husbands were full-sib or half-sib brothers and who sometimes had more than one wife. Thus, within each household, the children generally formed several full-sib, half-sib, and first cousin groups.

In study 2—the birth cohort study—we monitored the incidence of admission to hospital with malaria and other diseases through passive surveillance of a birth cohort of 2,914 children 5 y old or younger, residing in a wider geographical area that encompassed Ngerenya (study 1) within 16 km of Kilifi District Hospital between May 1992 and December 1997. The birth cohort was recruited from a continuous demographic surveillance system used to monitor child survival. For the purpose of this analysis, we classified admissions into three categories: (i) malaria, (ii) other infectious diseases (such as acute respiratory infections, meningitis, measles, or gastroenteritis), or (iii) accidents. In this paper we use the term “hospitalised malaria” to refer to hospital admissions with malaria, but do not distinguish between the severity of disease within this class (for example, cerebral malaria, severe malarial anaemia, or neither of these). In this study, we identified full-sibs, but did not record other information on genetic relationships.

### Data Analysis

For study 1, we calculated the annual incidence of malarial and nonmalarial fevers for each child separately as the number of episodes divided by the total number of weeks of surveillance, multiplied by 52. We performed this calculation both for each child over the entire period spent in the study (method 1), and for each age year the child spent in the study (for example as an 8-, 9-, and 10-y-old [method 2]). We excluded records for 3 wk following treatment for an episode of malaria. We also excluded data from patients with less than 30 records per year in order to standardise the measurement variation in incidence and, in the case of infants, to minimise the influence of maternally acquired immunity. Finally, we analysed data, including and excluding data from extreme children who suffered more than ten episodes of malaria or more than ten episodes of nonmalarial fever per year, on the basis that these were possibly manifestations of additional health problems. As the distribution of malaria incidence data was skewed, and because we expected heterogeneous variances across different age groups and years associated with their different means, we analysed our data both before and after applying 
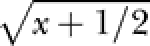
 or log_10_(*x* + 1) transformations.

We analysed our incidence data using a mixed linear model that incorporated genetic relationships to partition the total variation in disease incidence into its genetic, household, systematic, and other causes. The fixed effects fitted were age range (0–2, 3–5, 6–8, 9–10-y-olds, using the average for the entire study period; this effect was only fitted in method 1), year (one level for each year of study based on the average for the record in question), sex (male, female), use of mosquito nets over beds at night (yes, no, or unknown), ethnic group (Giriama, Chonyi, or other), and haemoglobin S genotype (wild type, *HbAA;* heterozygote, *HbAS*). The random effects fitted were for household and additive genetic value of each child. We separated additive genetic from other effects pertaining to an individual (nongenetic “permanent environmental,” or nonadditive genetic) by incorporating an “additive genetic relationship matrix” into our model [7]. This matrix, built from the pedigree list described above, contained the expected degree of genetic relationship between all children in the study (for example, ½ for full-sibs) and all their relatives. By incorporating this matrix into the model, the analysis essentially regresses the covariance among relatives in the trait under analysis onto their degree of genetic relatedness: this provides an estimate of the heritability based on all pairs of observations on related individuals from across the whole spectrum of relatedness. The model was fitted using restricted maximum likelihood procedures and the DFREML package [11].

We calculated the contribution of each of the fixed effects, and their sum (*f* ^2^), to the total variation in the trait (phenotypic variance, *V_P_*) from the ANOVA table, by dividing the type 3 sum of squares by the total sum of squares. Contributions of additive genetics (*h* ^2^) and household (*c* ^2^) were calculated from the ratio of their estimates of variance (*V_A_* and *V_C_*) to *V_P_.* Approximate standard errors of *h* ^2^ and *c* ^2^ were calculated from the information matrix around the maximum of the likelihood surface [12].

We analysed our data in two ways. First, we analysed the average incidence over the entire study period (1 record per child) using the above model (method 1). However, since different children spent different periods of time in the study, and because variances might differ across ages and years in association with their different means, we were concerned that the model's assumption of homogeneous errors would be violated. Therefore, we also performed a second analysis using repeated records on the same child, one for each full age year recorded (method 2), in which we fitted a multivariate model treating each age year as a separate, but correlated, trait (that is, 11 traits for age years 0–10). This method thus allowed *h* ^2^ and *c* ^2^ to vary across age groups: it also yielded estimates of genetic and environmental correlations in incidence between age groups. Estimates of *h* ^2^ and *c* ^2^ and their standard errors of annual incidence were pooled across age groups, using appropriate weights for the number of observations in each age group.

In study 2, our outcomes of interest were rates of admission to hospital with malaria, other infectious diseases, and accidents. We excluded data from children who were alive at the end of the study but who were absent from the district for more than 5 mo during the study period. We included data from children who died during their hospital stay, but excluded data from those who either died outside hospital or at less than 1 mo of age.

Given the binary nature of the data, we used threshold models for these analyses. These models assume that there is an underlying normally distributed “liability” trait, with a threshold level above which the disease is manifested. These took three forms. First (method 3), we compared the incidence of malaria admissions to hospital among affected full-sibs to that in the general, unrelated, population: a difference in incidence among relatives reflects a genetic component in the trait on the underlying scale and thus allows an estimation of its heritability [13]. This method does not allow for incorporation of fixed effects into the analysis, or for separation of *h* ^2^ from *c* ^2^, but does provide an easy way of calculating an upper limit to heritability from incidence data. In method 4, we analysed the data on the observed (binary) scale under a model fitting random effects for sibship and household, and fixed effects for age, sex, ethnic group, and bednet use. In this model, we did not fit year as it was heavily confounded with age in this study, and no information was available on haemoglobin S genotype. The estimates of *h* ^2^ and *c* ^2^ on the observed scale were then transformed to the underlying scale [14]. In method 5, we fitted the same model as in method 4, but data were analysed on the probit scale using general linear modelling procedures [15]. For method 3, data from all children were used in order to obtain an accurate estimate of incidence in the general population. However, in methods 4 and 5, data from children not in sibships were excluded because they contained no relevant information. Standard errors of *h* ^2^ and *c* ^2^ estimates were calculated based on method 4 [16].

To determine whether genetic and household effects could be adequately separated from the data structure encountered in these studies, we simulated phenotypic data according to the observed pedigree and household structure, assuming a range of values of additive genetic and household variances between 0.1 and 0.5, a phenotypic variance of 1, and either a normal (study 1) or binary distribution (study 2) of the trait. Ten replicate datasets per parameter combination were generated and then analysed under a model fitting household and an additive genetic effect per person as random effects.

## Results

The study design, and estimates of *h* ^2^, *c* ^2^ and *f* ^2^ from both these studies, and from a previous similar study conducted in Sri Lanka [2], are summarised in Table 1. As estimates in study 1 did not change by more than 0.05 as a result of transforming the data or excluding values of greater than ten episodes per year, only the estimates based on untransformed, uncensored data are shown.

**Table 1:**
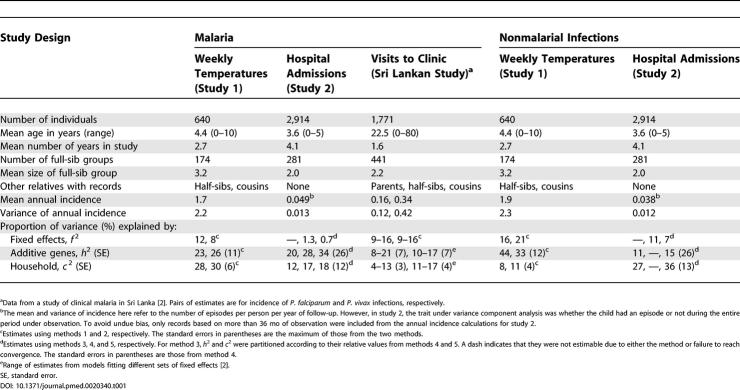
Estimates of the Contribution of Genetic, Household, and Other Effects to the Total Variation in Disease Incidence in the Present Studies in Coastal Kenya (Studies 1 and 2) and a Similar Study in Sri Lanka

### Study 1

The final analysis of the mild disease cohort study included data from 640 children living in 77 different households with a total of 1,727 annual age year records (2.7 per child). We identified the parents of 602 of these children (177 fathers, 222 mothers) and one or both grandparents of 119. The total pedigree included 1,590 individuals. An average household comprised 8.3 children fathered by an average of 2.3 men, themselves brothers, half-brothers, or first cousins, each with an average of 1.14 wives. Thus there were typically three groups of full-sibs per household who also formed groups of first cousins or half-sibs. The ethnic composition of this population was 84% Giriama, 10% Chonyi, and 6% other Mijikenda.

The incidence of nonmalarial fevers decreased rapidly from birth and averaged 1.9 episodes per child per year over the ages 0–10 y (Figure 1). In contrast, the incidence of malaria increased until age 3 y, but then remained stable until 10 y of age, the overall average being 1.6 episodes per child per year. Because superinfection (new infections in people who are already infected) is common in malaria, and the total number of fevers remained approximately constant between these ages, it is probable that our case definition more accurately represented the prevalence of blood-stage parasites than it did the incidence of new infections. Less than 5% of these infections resulted in hospital admission.

**Figure 1:**
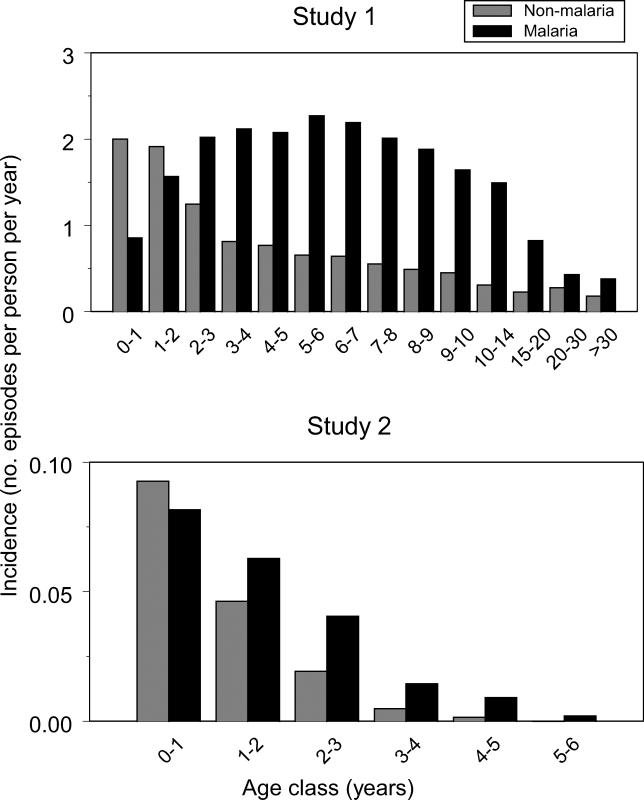
Mean Annual Incidence of Malarial and Nonmalarial Fevers in the Mild (Study 1) and Hospitalised (Study 2) Disease Studies in Coastal Kenya

In Figure 2, we have partitioned the total variation in the incidence of mild malaria and nonmalaria fevers, when averaged over the entire study period (method 1), into its components. As sex and bednet usage each explained less than 0.3% of this variation, we have omitted them from the figure. Ethnic group and *HbAS* each accounted for around 2% of the variation in malarial fevers and less than 0.4% in nonmalarial fevers, even when additive genetics and household were not included in the model. Age explained a lower proportion of the variation in malarial fevers than nonmalarial fevers, reflecting the age-incidence patterns for each (see Figure 1). Averaging the estimates from methods 1 and 2, additive genetics (*h* ^2^) explained 24(± 11)% and 39(± 12)% of the variation in malarial and nonmalarial fevers, respectively: the corresponding estimates for household effects (*c* ^2^) were 29(± 6)% and 9(± 4)%, respectively.

**Figure 2:**
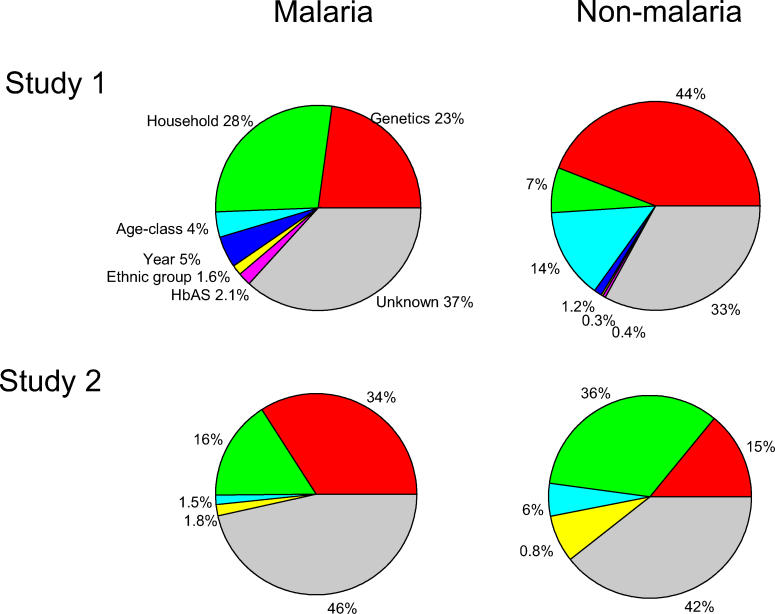
Proportions of Variation Explained by Additive Genetics, Household, and Other Factors in the Incidence of Malarial and Nonmalarial Mild (Study 1) and Hospitalised (Study 2) Disease in the Study Populations of Coastal Kenya Using Method 1 Colour coding for all four charts follows that in the top left using method 1.

Although phenotypic variances correlated positively with mean incidence at each age, there was no obvious change in *h* ^2^ with age. Genetic correlations between incidence at consecutive ages averaged 0.40 and 0.46 for malaria and nonmalaria, respectively. The corresponding “environmental” (residual) correlations were −0.01 and 0.02, and phenotypic correlations were 0.36 and 0.26. Phenotypic, genetic, and environmental correlations between the incidence of malarial and nonmalarial fevers within age years were −0.01, 0.23 and −0.29, respectively, the latter no doubt reflecting the fact that these two traits represent opposite sides of the same coin. When averaged over years, the corresponding values were 0.37, 0.61, and 0.38, suggesting that children share susceptibilities to both types of fever for both genetic and nongenetic (but not household) reasons. The mean correlations between age groups in household effects for malaria and nonmalaria were 0.81 and 0.55, respectively, indicating that household effects were consistent across age groups, especially for malaria.

### Study 2

During the study, 2,914 children remained resident, their average age at the end of the study or at death (and hence length of time under surveillance) being 4.1 y (range 1 mo–5.7 y). Overall, 33% of these children were admitted to the hospital at least once during the study period. Forty-eight percent of all admissions were due to malaria, while a further 26%, 9%, and 2% of admissions were due to acute respiratory infections, gastrointestinal infections, and accidents, respectively. The incidence of malaria and other infections decreased rapidly with age (Figure 1). The average incidence of hospitalised malaria over the 4.1 y was 0.054 per child per year (compared to 1.64 per year in children ≤5 y old in study 1). The average age at first admission with malaria was 1.6 y (range 5 wk–5.3 y), and the average age of all first admissions was 1.4 y (4 wk–5.3 y). Case-fatality rates for hospitalised malaria and other illnesses were 2.6% and 2.3%, respectively. The ethnic composition of the population in study 2 was broadly similar to that in study 1, being 77% Giriama, 13% Chonyi, and 10% other Mijikenda.

Estimates of *h* ^2^ and *c* ^2^ for nonmalarial infections in this study were respectively lower and higher than for malaria, reversing the pattern seen for mild infections in study 1 (see Figure 2; Table 1). On the other hand, in the case of both mild and hospitalised disease, fixed effects accounted for more of the variation in nonmalarial infections than in malaria. Our estimates of *h* ^2^, *c* ^2^ and *f* ^2^ for accidents were 0, 0, and 4%, respectively, although the incidence was too low for these to be reliable.

The simulation study showed that estimates of *h* ^2^ and *c* ^2^ were unbiased by the data structure (that is, by confounding between genetic groupings and household) but that, as expected from some confounding, the sampling correlations between them were −0.5 for study 1 and −0.7 for study 2. This means that if, for sampling reasons, the true *h* ^2^ was overestimated by 0.2 (that is, two standard errors), *c* ^2^ would be underestimated by 0.05 in study 1 and by 0.07 in study 2. These simulations also showed that our estimates of standard errors were reasonable.

## Discussion

Our analyses of data from two independent studies conducted on the coast of Kenya, and a third study conducted in Sri Lanka [2], each focussed on a different part of the wide spectrum of disease severity in malaria, and using a variety of statistical methods, suggest that host genetic factors generally account for about a quarter of the total variation in the susceptibility of individuals to malarial disease (Figure 2; Table 1). The Sri Lankan study differed from the Kenyan studies in that it had a much lower transmission intensity and hence disease incidence, none of which was severe, a high prevalence of *Plasmodium vivax* in addition to *P. falciparum,* a study population consisting mainly of adults, and an entirely different genetic composition to that of Kenya. Nevertheless, the results from both studies are in broad agreement, suggesting that substantial genetic variability for resistance to infection and disease severity is maintained in human populations that have been exposed to the disease for a very long time.

We could only attribute a small proportion of this variation to the best known of the malaria resistance genes, *HbS* and α-thalassaemia. For example, in theory [6] we expect that *HbAS,* which roughly halves the incidence of mild malaria (equivalent to half a standard deviation in incidence) and is found at a frequency of 0.15 in our population [17,18], would only explain around 2.5% of the total variation (2.4% additive genetic and 0.06% dominance variation) in incidence of mild clinical malaria, a figure close to that derived through observation in our studies (Figure 2). Similarly, we would anticipate that the mutant that causes α-thalassaemia*,* which is found in our population at an allele frequency of 0.43, and which reduces the incidence of uncomplicated malaria by around 0.4 of a standard deviation in homozygotes and a little more than half this in heterozygotes (T. N. Williams, personal communication), would account for only 2% (1.9% additive and 0.1% dominance) of the total phenotypic variation [6]. For hospitalised malaria, the corresponding values are 0.6% for *HbAS* [18] and 0.7% for α-thalassaemia [19]. Thus, on their own, even the most prominent of the known malaria resistance genes make only minor contributions to the total impact of host genetics. These examples highlight the fact that, as shown by an increasing number of studies conducted both in the field and in the laboratory [20–23], malaria resistance is under complex, multigenic control, with each individual gene having a relatively small epidemiological impact.

The heritability estimates we report here are almost certainly conservative for several reasons. First, we anticipate that our assessment of paternity will have been subject to error, reducing our observed relative to true estimates by a factor of (1 − *p*)^2^ where *p* measures the misclassification of paternity [24]. Second, our models only estimated the contributions of genes that act additively: the effects of genes with nonadditive effects, such as dominance or epistasis—the latter of which has already been demonstrated for some known resistance genes [25]—will not contribute to the heritability estimates reported here. Although statistical models are available that allow estimation of nonadditive genetic variance, much larger sets of suitably structured data would be required to obtain reliable estimates. Finally, there is growing evidence to suggest that variability in parasite virulence genes interacts with host genetic polymorphisms [26]: this form of host genetic variability is not represented in the additive genetic heritabilities estimated here.

Our studies suggest that host genetic factors also affect susceptibility to nonmalarial fevers and, to a lesser extent, nonmalarial infections leading to hospital admission. Indeed, the high genetic correlation between the average rate of malarial and nonmalarial fevers (61%) suggests that susceptibility to a range of childhood infections might be mediated via mechanisms with a common genetic basis. High heritabilities of the immune response to some antigens from malaria parasites and other infectious pathogens [3,27,28,] may indicate genetic control of a generalised immune response to pathogens. This does not rule out the possibility that some individual genes may have specific, but opposing effects on resistance to malaria versus other pathogens, thus perhaps helping to maintain the remarkable degree of genetic variation for disease resistance observed in this heavily selected population.

A striking result from this study was the amount of variation that could be attributed to household, particularly in the incidence of mild malaria and nonmalarial hospitalised infections. Children living in the 10% most malarious houses had about twice as many malaria infections (2.39 per year) as those in the 10% least malarious houses (1.14 per year). As our measure of malaria incidence almost certainly includes superinfections, this between-house variability probably reflects variation in transmission intensity due to spatial variation in breeding sites for mosquitoes and other household-related factors such as insecticide and repellant use [29]. We could not attribute the between-household variation in transmission intensity to bednet use, as also found in a second study in this area [29]. Even though untreated bednets in good condition are protective in this study area, damaged nets are not [30]. In the present analysis, where we considered whether or not bednets of any kind were being used, we did not find a protective effect. Socioeconomic factors such as quality of building and surrounds, nutrition, education, and access to health care may also play a part in explaining between-household differences in both malarial and nonmalarial infections. Clearly, identifying and improving factors relating to the risk of individual households would go a long way towards relieving the burden of disease in children living under such conditions.

This study shows that despite the inherent stochasticity in malaria transmission, the average risk of malaria in children living in our study area is in large part due to factors that are predetermined, both at the genetic and nongenetic level. While manipulation of the host's genes or their products may not yet seem plausible, determining how specific genes control the protective response may, ultimately, lead to a better understanding of the mechanisms of pathogenesis and host resistance. In the meantime, tackling household-related factors would seem to be a more tractable option for disease control.

**Patient Summary**

**Background**

Humans exposed to malaria get infected and sick to varying degrees, and some of that variation is due to differences in genetic makeup between individuals. Because the disease has killed humans for thousands of years, selection over time has increased protective variants of human genes in regions where malaria was and is common. One well-known example is the sickle-cell variant of the hemoglobin gene, which protects against severe malaria and is more common in people of African descent.

**Why Was This Study Done?**

Most research has focused on identifying the specific genes whose variants confer susceptibility to or protection from malaria. Some have been identified, but it is also clear that there are many genes involved, most of which contribute a small amount to the overall picture. In this study, the researchers wanted to estimate how much all genetic factors taken together, relative to the many environmental factors that also affect malaria risk, influence the number and severity of malaria cases.

**What Did the Researchers Do and Find?**

The researchers recruited and studied groups of children in rural Kenya. To estimate the overall contribution of genetic factors, they needed to observe the children over a long enough time that they would get a sense of an individual's malaria risk. In addition, they needed to know the genetic relatedness among the children, and the living arrangements needed to be such that children of different degrees of genetic relatedness (full siblings, half siblings, first cousins, etc.) shared a common environment. They found that genetic differences among people accounted for about 25% of the variability in malaria risk. This was less than the contribution by “household factors,” which accounted for around 30% of the total variability. Some of these household factors are known ones, such as insecticide use, but most of the 30% in risk variability was due to unidentified household factors.

**What Do These Findings Mean?**

The risk of malaria is strongly predetermined by genetic and nongenetic factors. However, while understanding how specific genes affect malaria risk will ultimately lead to a better understanding of the disease and improve prevention and treatment, genetic factors are not the biggest contributors to malaria. Therefore, in the short term, focusing on identifying and improving household-related factors is more likely to reduce the burden from the disease.

**Where Can I Get More Information Online?**

General information on the disease from the World Health Organization and links to many other sites:

http://www.who.int/malaria

The Wellcome Trust's malaria pages, which include a section on malaria and people that discusses genetic factors:

http://www.wellcome.ac.uk/en/malaria/home.html

The malaria pages of the Centers for Disease Control and Prevention, which contain a section on geographic distribution and epidemiology:

http://www.cdc.gov/malaria/distribution_epi/human_epidemiology.htm
